# The impact of surface treatment in 3-dimensional printed implants for early osseointegration: a comparison study of three different surfaces

**DOI:** 10.1038/s41598-021-89961-3

**Published:** 2021-05-17

**Authors:** Jungwon Lee, Jun-Beom Lee, Junseob Yun, In-Chul Rhyu, Yong-Moo Lee, Sung-Mi Lee, Min-Kyu Lee, Byoungkook Kim, Pangyu Kim, Ki-Tae Koo

**Affiliations:** 1 One-Stop Specialty Center, Seoul National University Dental Hospital, Seoul, Republic of Korea; 2 Department of Periodontology and Dental Research Institute, School of Dentistry, Seoul National University, 101, Daehak-ro, Jongno-gu, Seoul, 03080 Korea; 3 Department of Materials Science and Engineering, Seoul National University, Seoul, Republic of Korea; 4 Biomedical Implant Convergence Research Center, Advanced Institutes of Convergence Technology, Suwon, Korea; 5 3D Printer R&D Team, Dentium Co., Ltd., Suwon, Republic of Korea

**Keywords:** Medical research, Preclinical research

## Abstract

3D printing technology has been gradually applied to various areas. In the present study, 3D-printed implants were fabricated with direct metal laser sintering technique for a dental single root with titanium. The 3D implants were allocated into following groups: not treated (3D-None), sandblasted with a large grit and acid-etched (3D-SLA), and target-ion-induced plasma-sputtered surface (3D-TIPS). Two holes were drilled in each tibia of rabbit, and the three groups of implants were randomly placed with a mallet. Rabbits were sacrificed at two, four, and twelve weeks after the surgery. Histologic and histomorphometric analyses were performed for the evaluation of mineralized bone-to-implant contact (mBIC), osteoid-to-implant contact (OIC), total bone-to-implant contact (tBIC), mineralized bone area fraction occupancy (mBAFO), osteoid area fraction occupancy (OAFO), and total bone area fraction occupancy (tBAFO) in the inner and outer areas of lattice structure. At two weeks, 3D-TIPS showed significantly higher inner and outer tBIC and inner tBAFO compared with other groups. At four weeks, 3D-TIPS showed significantly higher outer OIC than 3D-SLA, but there were no significant differences in other variables. At twelve weeks, there were no significant differences. The surface treatment with TIPS in 3D-printed implants could enhance the osseointegration process in the rabbit tibia model, meaning that earlier osseointegration could be achieved.

## Introduction

An additive manufacturing process, three-dimensional (3D) printing technology, has been gradually applied to various areas, including dental devices, due to its inherent ability of individualized design and production, which can fulfil the demand of precision medicine^1–3^. Recently, attempts have been made to apply this 3D printing technology to dental implants, and 3D-printed titanium material has shown biocompatible performance, opening up the possibilities of clinical applications^4^.

Among various metal 3D printing process, direct metal laser sintering (DMLS) is known to be efficient in fabricating complex geometry using a layer by layer manufacturing method^4^. The field of application of DMLS is gradually expanding due to its low cost, less waste of powder, and flexibility of creating complex 3D structure and materials^5^. With DMLS technology, various parameters including the porosity, pore interconnectivity, size, shape, and distribution and 3D structure of the implant can be handled in manufacturing the implants^6^. Recent study has reported that 3D printed dental implants was successfully manufactured, showing biocompatibility in vivo study^7^.

It is expected that immediate placement is more likely to be indicated due to the root divergence or convexity/concavity in 3D-printed implant placement. Although 3D-printed implants, displaying inherent rough surfaces, can improve osteogenic differentiation and early osseointegration following implantation compared with conventional machined surfaces^8^, secondary stability should be facilitated to achieve immediate or early loading protocol with 3D-printed implants. In addition, compromised osteogenic situations including osteoporosis or diabetes requires the promotion of osseointegration. Therefore, it is necessary to promote osseointegration to reduce the decrease in stability of the 3D-printed implant with implant surface modification.

Various implant surface modification techniques have been introduced and studied to promote osseointegration of dental implants^9–11^. Sandblasting with large grit and acid-etched (SLA) surface is one of the representative surface treatments to imbue proper roughness and has been proven in numerous literatures to be successful bone-to-implant contact^12,13^. In the context of surface treatment, a recent study reported that 3D-printed implant with SLA surface treatment showed an improved osteogenic differentiation of bone marrow-derived mesenchymal stromal cells and osseointegration in a rat model^14^.

Meanwhile, target-ion-induced plasma sputtering (TIPS) has been applied to fabricate large-scale, self-assembled nanopatterns on titanium surfaces^15^. The incorporation of the target material, tantalum, during the TIPS process has been utilized due to its high corrosion resistance and good biocompatibility^16^. Hierarchical micro-nano-structured surfaces of implants with TIPS application showed enhanced hydrophilicity and osteoblastic responses^17^. This led us to hypothesize that implants treated with TIPS could enhance the osseointegration process. Therefore, the aim of this study is to investigate the biologic performance of 3D-printed implants without surface treatment, with SLA, and with TIPS surface treatment in vivo. The null hypothesis is that there is no difference among the osseointegration levels of the three different surfaces.

## Results

### Surface characteristics and surface roughness measurement

**Figure 1:**
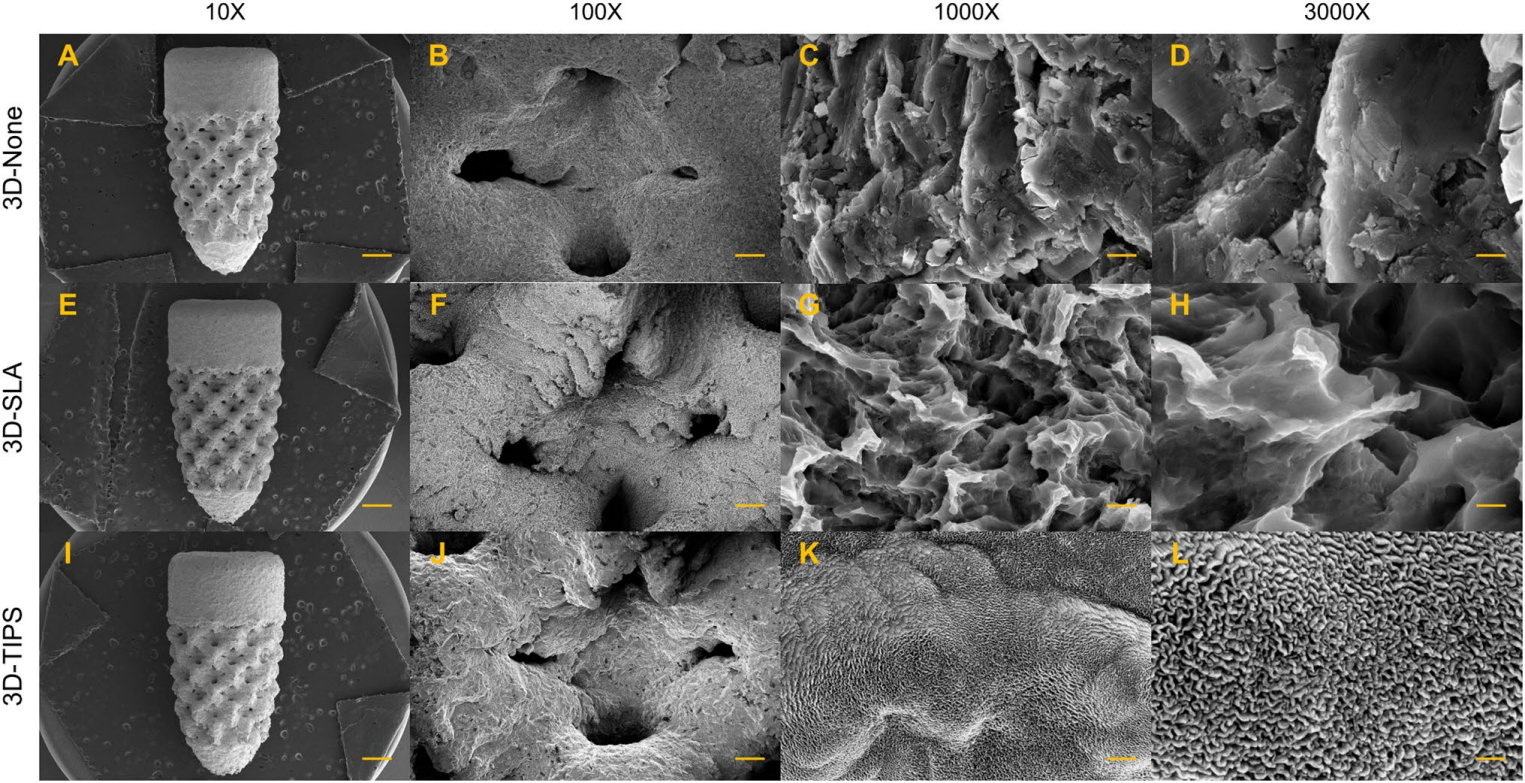
Scanning electron microscopy (SEM) images of 3D-printed implants. (**A**–**D**) 3D-printed implants without any surface treatments (3D-None). (**E**–**H**) 3D-printed implants sandblasted with large grit and acid-etching (3D-SLA), (**I**–**L**) 3D-printed implants with target-ion-induced plasma sputtering (3D-TIPS). Magnification: × 10 (**A**,**E**,**I**), × 100 (**B**,**F**,**J**), × 1000 (**C**,**G**,**K**), × 3000 (**D**,**H**,**L**) from the left to the right. Scale bar: 1 mm (10X), 100 μm (100X), 1 μm (1000X), 300 nm (3000X).

The surface topographies of 3D-None, 3D-SLA, and 3D-TIPS implants are presented in Fig. 1. 3D-None surfaces showed irregular patterns of empty space with several partly fused grains interspersed on the surface. 3D-SLA displayed lots of micron-submicron pits on the surface, consisting of a combination of large cavities 2–5 μm in size and small pits 300–900 nm in size without any fused grains of titanium. 3D-TIPS exhibited uniform ripple features with an approximately 50-nm gap width.

**Figure 2:**
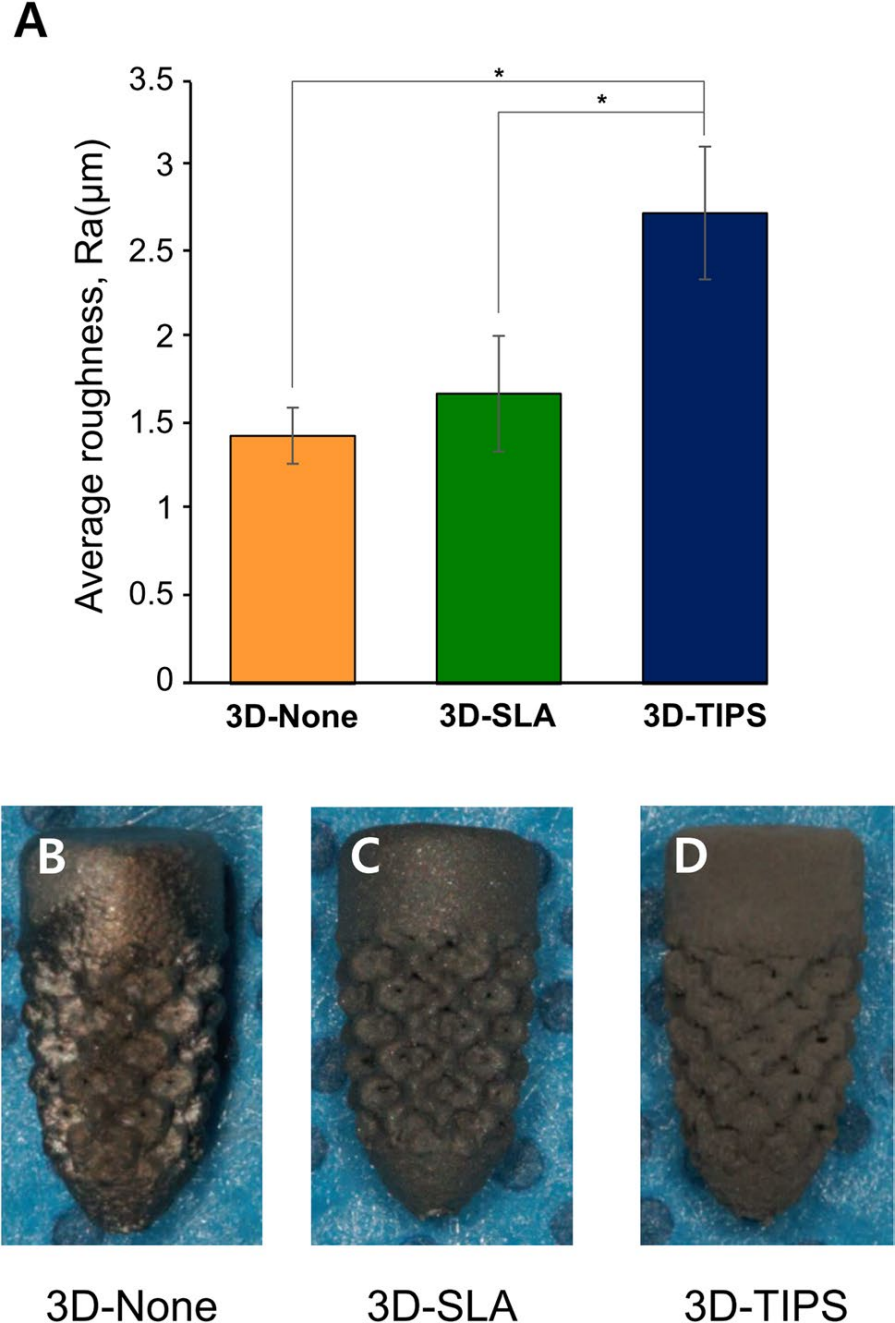
Quantitative analysis for surface roughness, Ra (μm). (**A**) The Ra of 3D-TIPS was significantly higher than in other groups. More grayish color was shown in ascending order from 3D-None (**B**), 3D-SLA (**C**), to 3D-TIPS (**D**).

The average roughness (Ra) values of 3D-None, 3D-SLA and 3D-TIPS were 1.44 ± 0.16 μm, 1.69 ± 0.34 μm, and 2.73 ± 0.39 μm, respectively (Fig. 2). There was no statistically significant difference between 3D-None and 3D-SLA in the value of Ra, while the Ra of 3D-TIPS was higher than both 3D-None and 3D-SLA.

### Histologic observation

Pathologic events, including inflammation or infection, were not observed in all specimens. Of a total of 27 animals, one in the 4-week observation group and one in the 12-week observation group died before the scheduled sacrifice date, and another one in the 12-week observation group showing abnormal movement due to severe pain and poor feeding after surgery was euthanized. Therefore, nine animals of twelve 3D-none, twelve 3D-SLA, and twelve 3D-TIPS in the 2-week observation group, eight animals of eleven 3D-none, ten 3D-SLA, and eleven 3D-TIPS in the 4-week observation group, and seven animals of ten 3D-none, ten 3D-SLA, and eight 3D-TIPS in the 12-week observation group were analyzed.

#### 2-week healing

**Figure 3:**
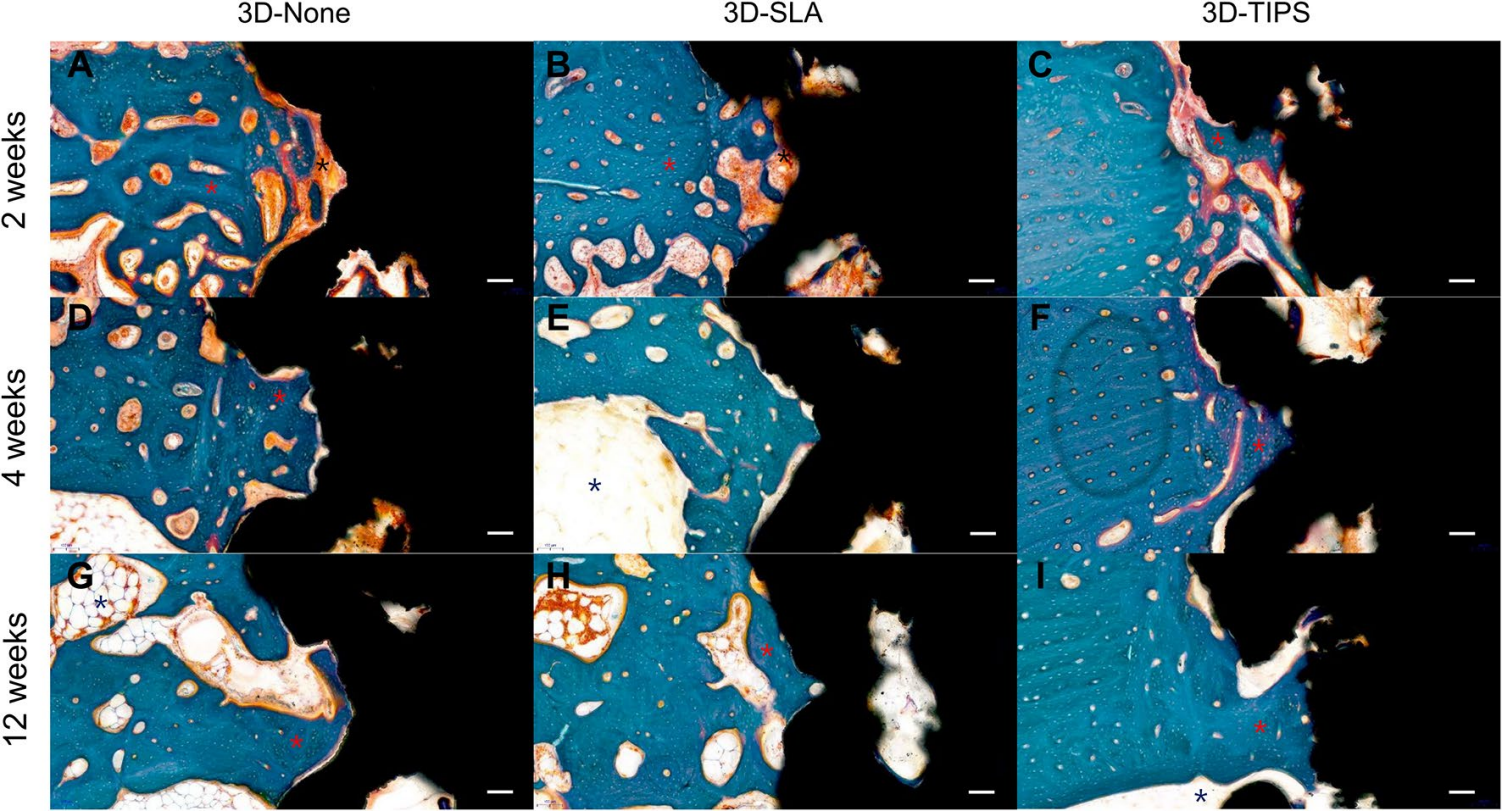
Representative histologic section with 10X magnification in 3D-None at 2 weeks (**A**), 4 weeks (**D**), and 12 weeks (**G**), 3D-SLA at 2 weeks (**B**), 4 weeks (**E**), and 12 weeks (**H**), and 3D-TIPS at 2 weeks (**C**), 4 weeks (**F**), and 12 weeks (**I**). Colored asterisks indicate mineralized bone (red), osteoid (black) and bone marrow (blue). Scale bar: 100 μm.

**Figure 4:**
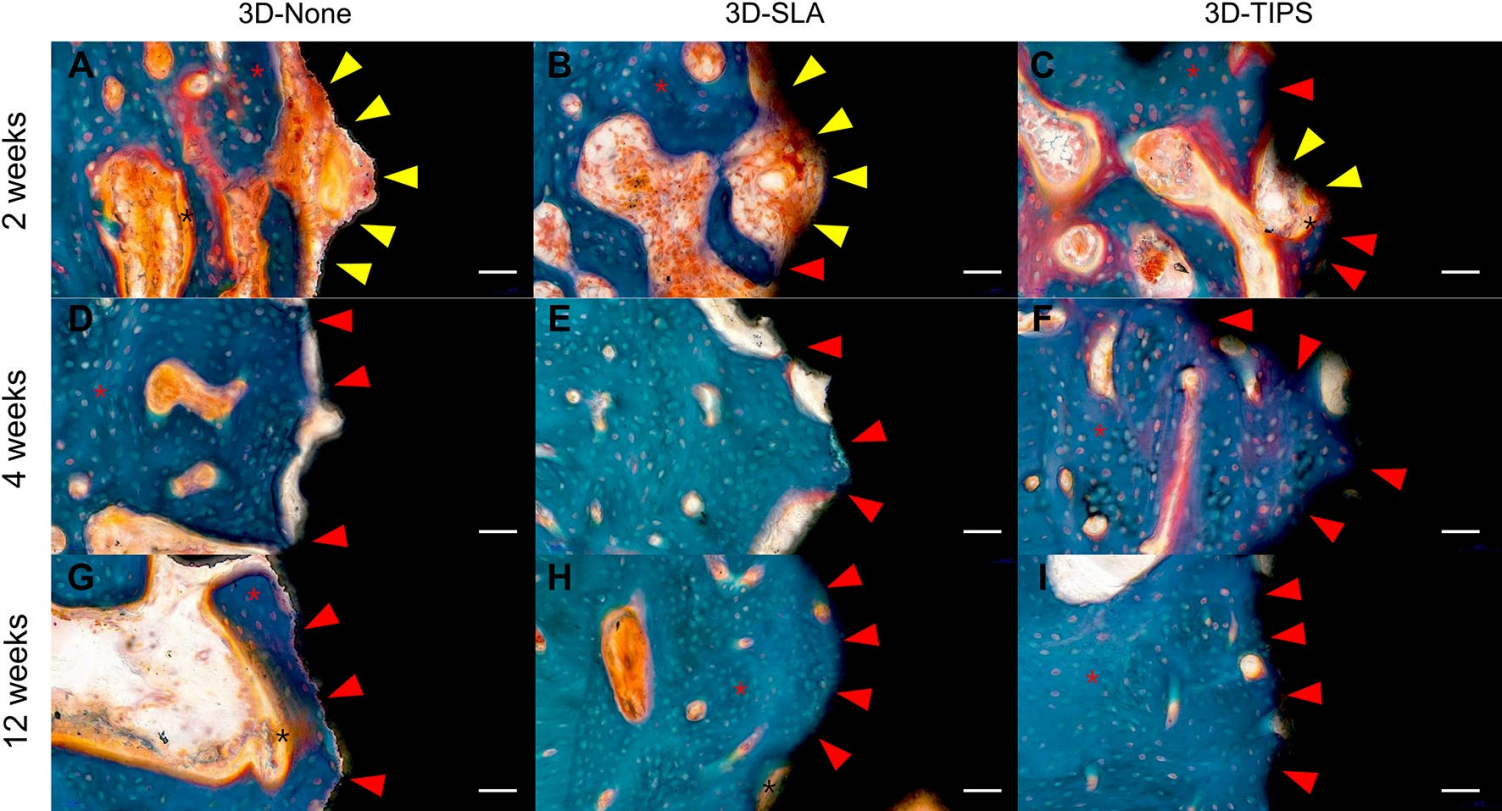
Representative histologic section with 30X magnification in 3D-None at 2 weeks (**A**), 4 weeks (**D**), and 12 weeks (**G**), 3D-SLA at 2 weeks (**B**), 4 weeks (**E**), and 12 weeks (**H**), and 3D-TIPS at 2 weeks (**C**), 4 weeks (**F**), and 12 weeks (**I**). Osteoid-to-implant contact (yellow arrow head) and bone-to-implant contact (red arrow head) were observed. Colored asterisks indicate mineralized bone (red) and osteoid (black). Scale bar: 300 μm.

Woven bone was formed at the upper and/or lower area of the cortical bone. New bone formation was shown besides the pristine bone area and regenerated toward the implant surface (Fig. 3). Meanwhile, randomly situated new bone formation was observed in the gap between the 3D-printed implant and pristine bone. Dynamic new bone formation was indicated with the existence of osteoid matrix (Fig. 4). In 3D-none, the proportion of osteoid was somewhat higher than that in 3D-SLA or 3D-TIPS.

#### 4-week healing

Newly formed bone mixed with woven and lamellar bones was extended from the osteotomy region to the implant surface (Figs. 3). More increased mineralized bone was observed in the spaces within the lattice structure of 3D-printed implants (Figs. 4). In contrast to the rich amount of osteoid area observed at 2 weeks of healing, more established bone formation was discovered. Matrix between the bone, with copious vascular structures, was also observed.

#### 12-week healing

Primary bone remodeling had nearly desisted and secondary remodeling was ongoing around all types of 3D-printed implants (Fig. 3). More active remodeling was observed at the adjacent interface of 3D-printed implants than at the area far from the surface (Fig. 4). The osteoid area was markedly reduced compared to the areas with 2- and 4- week healing, with intensive cement lines reflecting secondary osteon formation and lamellar bone deposition.

### Histomorphometric analysis

The mBIC, OIC, tBIC, mBAFO, OAFO, and tBAFO are presented in Tables 1, 2 and Table 3. At 2 weeks, significantly higher inner and outer tBIC were observed in the 3D-TIPS group compared to 3D-none. The inner and outer mBIC and OIC showed higher value in the 3D-SLA and 3D-TIPS compared to 3D-none, however, there was no significant differences. Whole mBIC showed higher values in 3D-SLA and 3D-TIPS compared to 3D-none. In the case of inner tBAFO, Kruskal–Wallis test showed significance (P = 0.046), however, there were no significantly differences between two groups when performing Bonferroni multiple comparison test. At 4 weeks and 12 weeks, there were no statistical differences among three implants. The outer mBIC, OIC, tBIC, mBAFO, OAFO and tBAFO showed gradual values from 2 weeks of healing to 12 weeks of healing. On the other hand, the inner mBIC, OIC, and tBIC showed highest values at 4 weeks of healing and decreased values at 12 weeks of healing except 3D-TIPS, however, inner mBAFO, OAFO and tBAFO showed the highest values at 2 weeks of healing compared with 4 weeks or 12 weeks of healing.

**Table 1:** Histomorphometric analysis of two-week healing.

2 week	3D-none		3D-SLA		3D-TIPS		P-value
	n	Mean ± SD	n	Mean ± SD	n	Mean ± SD	
Inner mBIC	12	5.64 ± 3.30	12	10.79 ± 14.24	12	12.83 ± 6.86	0.072
Outer mBIC	12	12.39 ± 7.88	12	18.92 ± 9.49	12	21.82 ± 11.98	0.109
Whole mBIC	12	8.47 ± 3.44 ^a^	12	14.32 ± 10.88 ^ab^	12	16.52 ± 4.37 ^b^	< 0.001
Inner OIC	12	9.97 ± 4.63	12	12.71 ± 7.29	12	16.51 ± 9.07	0.148
Outer OIC	12	10.84 ± 7.02	12	13.23 ± 9.08	12	11.77 ± 7.39	0.782
Whole OIC	12	9.79 ± 3.51	12	12.82 ± 6.60	12	14.00 ± 3.97	0.056
Inner tBIC	12	15.61 ± 5.55 ^a^	12	23.50 ± 13.48 ^ab^	12	28.72 ± 8.37 ^b^	0.004
Outer tBIC	12	23.23 ± 9.23 ^a^	12	32.16 ± 12.04 ^ab^	12	33.59 ± 8.18 ^b^	0.031
Whole tBIC	12	18.26 ± 3.30 ^a^	12	27.15 ± 10.57 ^ab^	12	30.52 ± 3.34 ^b^	< 0.001
Inner mBAFO	12	13.45 ± 6.13	12	9.43 ± 7.13	12	13.14 ± 7.33	0.242
Outer mBAFO	12	18.78 ± 6.92	12	23.13 ± 10.81	12	25.48 ± 13.58	0.302
Whole mBAFO	12	16.29 ± 5.95	12	16.57 ± 9.11	12	20.28 ± 10.21	0.405
Inner OAFO	12	7.85 ± 5.49	12	6.89 ± 6.97	12	10.25 ± 8.03	0.442
Outer OAFO	12	5.79 ± 3.89	12	8.64 ± 7.16	12	6.90 ± 3.89	0.688
Whole OAFO	12	6.59 ± 3.25	12	7.24 ± 5.85	12	8.31 ± 4.64	0.681
Inner tBAFO	12	21.18 ± 7.40 ^a^	12	14.92 ± 10.85 ^a^	12	23.39 ± 9.95 ^a^	0.046
Outer tBAFO	12	24.57 ± 7.70	12	31.77 ± 13.36	12	32.37 ± 13.82	0.282
Whole tBAFO	12	22.88 ± 6.10	12	23.81 ± 11.89	12	28.59 ± 11.57	0.224

Values are presented as mean ± standard deviation. mineralized bone-to-implant contact (mBIC), osteoid-to-implant contact (OIC), total bone-to-implant contact (tBIC), mineralized bone area fraction occupancy (mBAFO), osteoid area fraction occupancy (OAFO), and total bone area fraction occupancy (tBAFO). P-value by the nonparametric Kruskal–Wallis test. *Different letters, a and b, indicate statistical significance under Bonferroni correction (overall P- value < 0.05).

**Table 2:** Histomorphometric analysis of four-week healing.

4 week	3D-none		3D-SLA		3D-TIPS		P-value
	n	Mean ± SD	n	Mean ± SD	n	Mean ± SD	
Inner mBIC	11	14.99 ± 8.46	10	14.54 ± 8.31	11	10.34 ± 7.44	0.450
Outer mBIC	11	21.41 ± 10.55	10	29.78 ± 12.01	11	28.33 ± 7.53	0.051
Whole mBIC	11	17.79 ± 6.57	10	22.05 ± 8.92	11	18.64 ± 3.08	0.531
Inner OIC	11	14.94 ± 9.47	10	16.08 ± 9.04	11	13.57 ± 5.67	0.784
Outer OIC	11	11.39 ± 9.36	10	5.91 ± 5.50	11	11.88 ± 6.47	0.045
Whole OIC	11	13.34 ± 7.32	10	10.89 ± 5.46	11	12.69 ± 4.50	0.850
Inner tBIC	11	29.93 ± 9.57	10	30.63 ± 11.00	11	23.90 ± 8.74	0.182
Outer tBIC	11	32.80 ± 17.30	10	35.68 ± 14.60	11	40.21 ± 7.57	0.087
Whole tBIC	11	31.13 ± 9.53	10	32.94 ± 10.26	11	31.34 ± 6.24	0.804
Inner mBAFO	11	6.33 ± 6.79	10	3.09 ± 3.96	11	7.37 ± 5.43	0.075
Outer mBAFO	11	28.68 ± 20.22	10	24.20 ± 18.44	11	36.43 ± 17.60	0.325
Whole mBAFO	11	18.32 ± 13.69	10	13.92 ± 11.77	11	29.11 ± 12.89	0.266
Inner OAFO	11	10.38 ± 7.75	10	9.53 ± 10.82	11	9.00 ± 4.70	0.670
Outer OAFO	11	3.52 ± 3.23	10	3.81 ± 4.47	11	4.97 ± 5.79	0.624
Whole OAFO	11	6.70 ± 4.77	10	6.46 ± 5.33	11	6.66 ± 4.06	0.909
Inner tBAFO	11	16.71 ± 10.96	10	12.61 ± 14.07	11	16.38 ± 7.64	0.247
Outer tBAFO	11	32.20 ± 22.45	10	28.01 ± 20.30	11	41.40 ± 19.16	0.301
Whole tBAFO	11	25.02 ± 16.44	10	20.38 ± 14.45	11	29.11 ± 12.89	0.308

Values are presented as mean ± standard deviation.

**Table 3:** Histomorphometric analysis of twelve-week healing.

12 Week	3D-none		3D-SLA		3D-TIPS		P-value
	n	Mean ± SD	n	Mean ± SD	n	Mean ± SD	
Inner mBIC	10	9.43 ± 5.29	10	15.02 ± 7.95	8	15.37 ± 9.89	0.158
Outer mBIC	10	33.18 ± 13.21	10	31.25 ± 7.92	8	36.81 ± 12.97	0.8054
Whole mBIC	10	21.50 ± 8.04	10	22.24 ± 5.75	8	25.83 ± 10.26	0.772
Inner OIC	10	13.27 ± 22.98	10	8.75 ± 3.94	8	8.50 ± 5.64	0.543
Outer OIC	10	18.67 ± 9.25	10	16.75 ± 16.86	8	19.98 ± 11.39	0.480
Whole OIC	10	16.55 ± 10.41	10	12.37 ± 7.69	8	14.21 ± 6.79	0.539
Inner tBIC	10	22.70 ± 22.83	10	23.76 ± 9.89	8	23.88 ± 11.73	0.955
Outer tBIC	10	51.85 ± 9.72	10	48.00 ± 19.31	8	56.79 ± 13.30	0.126
Whole tBIC	10	38.05 ± 13.35	10	34.62 ± 8.83	8	40.04 ± 10.69	0.539
Inner mBAFO	10	8.03 ± 6.47	10	7.89 ± 4.82	8	10.15 ± 9.69	0.910
Outer mBAFO	10	34.35 ± 24.68	10	18.41 ± 7.42	8	36.71 ± 22.79	0.252
Whole mBAFO	10	23.20 ± 15.29	10	13.19 ± 5.95	8	26.40 ± 18.13	0.296
Inner OAFO	10	6.31 ± 9.38	10	3.73 ± 2.50	8	4.66 ± 3.75	0.903
Outer OAFO	10	4.80 ± 2.79	10	3.04 ± 1.95	8	2.52 ± 2.06	0.121
Whole OAFO	10	5.67 ± 4.57	10	3.28 ± 1.90	8	3.52 ± 2.15	0.409
Inner tBAFO	10	14.33 ± 10.77	10	11.62 ± 6.45	8	14.81 ± 10.76	0.792
Outer tBAFO	10	39.15 ± 23.63	10	21.45 ± 7.49	8	39.23 ± 23.44	0.206
Whole tBAFO	10	28.87 ± 13.99	10	16.47 ± 6.27	8	29.92 ± 18.89	0.146

Values are presented as mean ± standard deviation.

## Discussion

In this study, we found that surface treatment of 3D printed implants might facilitate early deposition of organic matrix and mineralized bone, which is more pronounced in 3D-TIPS, meaning that surface treatment in 3D-printed implants could result in a faster osseointegration process. At 12 weeks, the three surface treatments were not significantly different, indicating that similar levels of osseointegration could be achieved regardless of surface treatment modalities if implants have a sufficient healing period.

The surface modification technology in implant dentistry was developed by altering the surface topography from machined to rough surface of titanium because roughened surface has been shown to improve the osteoblastic differentiation and the calcium deposition and osseointegration levels in in vitro and in vivo studies^18^. At 12 weeks, all the groups showed similar levels of osseointegration. 3D-printed implants have an inherent roughness, in this study showing a 1.45-μm Ra value without any surface treatments. 3D-None and 3D-SLA showed similar roughness levels. A systematic review suggested the roughness of the surface of threaded dental implants should be calculated according to the Ra value. An Ra of 1.45 μm is classified as moderately rough^19^, which is favorable for the osseointegration process. In addition, 3D-printed implants made by direct metal laser sintering technology showed a porous structure and similar bone-to-implant contact compared with threaded implants made by milling and SLA surface treatment^7^. 3D-TIPS showed the highest Ra value, but a roughness higher than 2 μm might not significantly improve the osseointegration process. In this study, after the 12-week healing period, the surfaces were not significantly different. Meanwhile, the interest in implant surfaces has now moved on to how to load the implants earlier^20^. To do this, the implants should be anchored faster with the bone mechanically and biologically, with a sufficient level to bear the occlusal force with 200–500 N. In this respect, TIPS could be a favorable method, which achieved higher BIC in this study compared with other surface modifications.

The TIPS technique has another important feature in addition to roughening a titanium surface. A previous study showed that TIPS could provide a nanoporous structure on a titanium surface, which could act as a carrier for biomolecules such as recombinant human bone morphogenetic protein-2 (rhBMP-2)^21^. The titanium treated with TIPS showed a significantly higher level of rhBMP-2 in terms of loading capacity and release. This feature might be feasible in compromised osteogenic situations, such as osteoporosis or diabetes^21,22^.

The implant design used in this experiment has a lattice structure in the middle part. The lattice structure was reported to increase the BIC ratio and osteoblastic activity^7^. In this study, we analyzed the BIC, OIC, BAFO and OAFO in the inner and outer parts of the lattice structure, respectively. The measurement was needed to evaluate how much each surface attracted bone growth in this complicated lattice structure. Though the results were not significant, there was some trend that TIPS showed better and higher bone growth. Nanoscale modification in implant surface showed increase of wettability, resulting in improved osteogenic cell behaviors^23^. It has been demonstrated that bone tissue is intermixed with the TiO_2_ layer on the surface of the implant, and mineral platelets and collagen fibers can be found on the TiO_2_ layer^24^. The increased trend of osteogenic activity might be due to increased wettability and relatively uniform nanostructure in TIPS for intermixing bone tissue and TiO_2_ layer.

We performed a malleting for placement of 3D-printed implants after drilling a hole with a proper diameter in the bone. This was because we could only make a cylindrical hole with commercially available drilling tools, but the shape of the 3D-printed implants exactly mimics the tooth root. This procedure might reduce the mechanical stability of 3D implants. More feasible tools for the placement of 3D printed implants should be developed. Furthermore, malleting procedure may raise an issue of surface structure deformation. It is necessary to fabricate an implant with increased strength materials which guarantee surface structure stability following the implant placement with malleting method.

In this study, we did not evaluate the 3D-printed implants under the loading condition, mimicking more real clinical situations. Occlusal force creates stress on the osseointegrated bone around implants, and bone could respond differently to mechanical stress. Therefore, this aspect should be considered in future studies.

In conclusions, the surface treatment with TIPS in 3D-printed implants could enhance the osseointegration process in the rabbit tibia model, meaning that earlier osseointegration could be achieved. However, after a sufficient healing period (twelve weeks), 3D-printed implants could achieve similar levels of osseointegration regardless of surface treatment modalities.

## Methods

**Figure 5:**
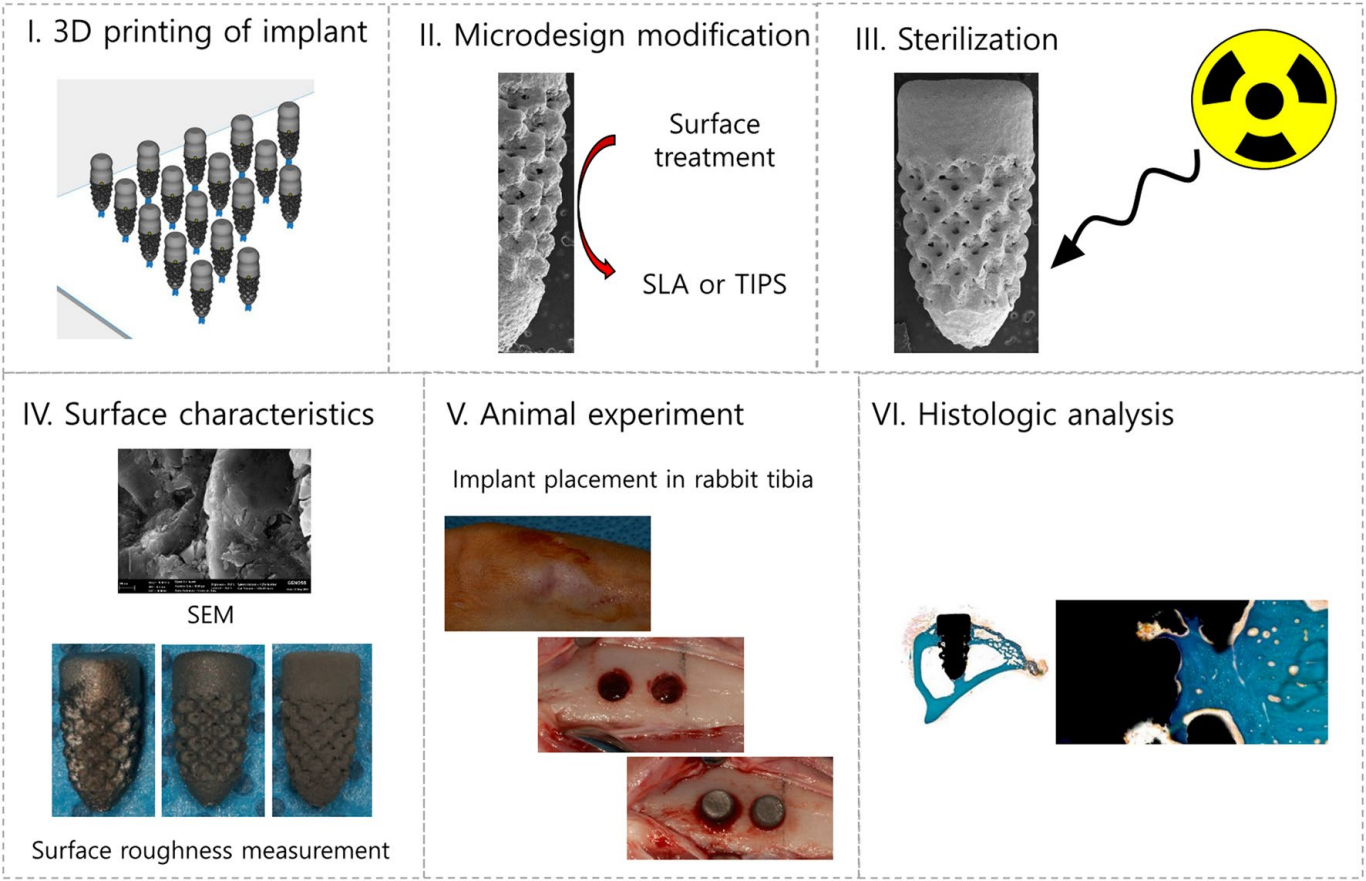
Experimental procedure in this study. The implants were fabricated through three-dimensional printing techniques, and then the surface treatments were performed in the experimental groups: 3D-SLA (sandblasted with large grit and acid-etched) and 3D-TIPS (target-ion-induced plasma sputtering). The control group was not treated (3D-None). Subsequently, all implants were cleaned and sterilized with gamma irradiation. Two implants were placed in each rabbit tibia. After sacrifice, histologic and histomorphometric analyses were performed.

The overall procedures in this study are summarized in Fig. 5.

### Fabrication of 3D-printed implants

3D-printed implants were fabricated with commercially available titanium grade 2 powder (Concept Laser GmbH, GE Addictive, Lichtenfels, Germany) and a laser printing device (Mlab200R, Concept Laser GmbH, GE Addictive, Lichtenfels, Germany). The particle size distribution of titanium powder were D_10_ = 20.53 μm, D_50_ = 35.87 μm, and D_90_ = 60.62 μm. The theoretical densities (ρ_theo_) was 4.5 g/cm^3^. The processing parameters are shown in Supplement 1. A single root form shape of 3D-printed implants that was 3.8 mm in diameter and 10 mm in length was prepared for this experiment. The middle part of the root area was fabricated with a lattice structure to increase the bone-to-implant contact area.

Following thorough cleansing with sequential ultrasonic cleansing in vacuum and subsequent drying of 3D-printed implants, all 3D-printed implants were allocated into 3 groups: the 3D-printed implant with no surface treatment group, the 3D-printed implant with SLA surface treatment group, and the 3D printed implant with TIPS surface treatment group. The SLA treatment were performed with sand blasting with alumina oxide particles and acid etching with hydrochloric acid and nitric acid Then, residual acid and debris was removed by ultrasonic cleaning and high pressure washer. TIPS surface treatment was performed by target-ion-induced plasma sputtering according to a previous study^15^ directly on the 3D printed implant. The fabricated implants were cleansed through six consecutive procedures: four ultrasonic cleansing under vacuum conditions for 8–10 min and subsequent hot air drying and vacuum drying. It was sterilized by irradiation with gamma ray, which is a short wavelength light emitted from the Cobalt-60 (^60^Co) radioactive isotope according to ISO11137.

### Surface characteristics evaluation

The surface topographies of the three groups of 3D-printed implants were observed using a scanning electron microscope (Gemini SEM 300, Carl Zeiss, Oberkochen, Germany) following gold coating.

For the quantitative evaluation of the surface roughness of the 3D-printed implants with different surface treatments, a surface roughness tester (SJ-412, Mitutoyo, Japan) was used for surface roughness parameter measurement according to the previous study^25^. The measurement conditions were as follow: Stylus: 1.2, Measurement Length: 0.75 mm, Speed: 0.05 mm/s, and Filter: Gaussian (according to ISO 1997). The surface roughness was measured only on the upper part of the implant and lattice area was not measured. Five samples were measured for each group.

### Animal experiment

Animal experiment was performed in accordance with the principles of the 3Rs (Replacement, Reduction, and Refinement) and two main laws in Korea which are Animal Protection Act established by the Ministry of Agriculture Food and Rural Affairs, and the Laboratory Animal Act established by the Ministry of Food and Drug Safety. The animal experiment was reviewed and approved by the Institutional Animal Care and Use Committee of Seoul National University (IACUC; approval no. SNU-190619-3-1) and conducted in accordance with the ARRIVE guidelines. The tibial model in rabbit was used to evaluate the biologic responses of 3D-printed, 3D-printed with SLA and 3D-printed with TIPS implants. The rabbit tibial model was used because the aim of this study was to investigate the biologic performance of 3D-printed implants without surface treatment, with SLA, and with TIPS surface treatment in the absence of mechanical loading according to the previous studies^26,27^. Nine rabbits each were assigned to the 2-week group, 4-week group, and 12-week group, and a total of 27 rabbits were involved in this study. Rabbits weighing approximately 3 ~ 4 kg (DooYeol Biotech, Seoul, Korea) were given general anesthesia with Zoletil (7.5 mg/kg, Virbac, Carros, France) and Rompun (3.5 mg/ml, Bayer Korea, Gyeonggi-do, Korea) intravenously, and the surgical sites were shaved and disinfected with povidone-iodine. After an incision was made at the proximal metaphyseal-diaphyseal area of the tibia, the full-thickness flap was reflected, and the periosteum was gently raised to expose the implantation position. Subsequently, surgical drills (Dentium, Gyeonggi-do, Korea) were used to make implant preparations of two 3.35-mm-diameter holes per tibia with 1,000 rpm and 45 N·cm. 3D-printed implants with different surfaces were inserted with a mallet. The type of inserted 3D-printed implant was allocated randomly with a predetermined sequence using the web site http://www.randomization.com. The tissues were sutured in layers with 4/0 vicryl (Ethicon, Somerville, New Jersey, USA) and 5/0 monosyn (B.Braun, Melsungen, Germany) (Supplement 2). All rabbits received antibiotics and analgesics for 3 days to prevent infection and to relieve any pain. The animals were checked daily for adverse reactions or abnormal behavior.

### Biopsy and histological processing

The animals were deeply anesthetized with Zoletil and Rompun and euthanized by means of intravenous injection of potassium chloride (0.15 g/ml, Jeil, Seoul, Korea) at two, four, and twelve weeks following implant placement. The tibia of each rabbit was extracted, and the soft tissues were removed. Hard tissue including the implants was fixed in a buffered neutral formalin solution (Sigma-Aldrich, St. Louis, Missouri, USA) for two weeks and was subsequently dehydrated in graded ethanol solution. Thereafter, the samples were embedded in resin blocks (Technovit 7200; Heraeus Kulzer, Wehrheim, Germany) with a UV embedding system (KULZER EXAKT 520, Germany) according to the manufacturer’s recommendation. The sectioning procedure was implemented using a diamond saw and grinding system; thereafter, the final tissue section was polished to 40 ± 5 μm in thickness using an EXAKT grinding system (KULZER EXAKT 400CS, Germany). The samples were stained by Goldner trichrome.

### Histologic and histomorphometric analysis

**Figure 6:**
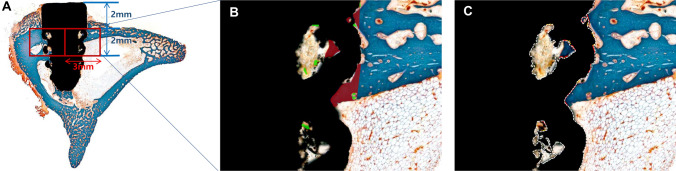
(**A**) The implants were demarcated with inner, outer, and whole areas at the region of interest (ROI) due to the lattice structure of the 3D-printed implants. (**B**) Colored areas indicate mineralized bone (red) and osteoid (green). Mineralized bone area fraction occupancy (mBAFO), osteoid area fraction occupancy (OAFO), and total bone area fraction occupancy (tBAFO) were measured. (**C**) Colored dotted lines indicate 3D-printed implant surface (white), mineralized bone-to-implant contact (red), and osteoid-to-implant contact (orange). Mineralized bone-to-implant contact (mBIC), osteoid-to-implant contact (OIC), and total bone-to-implant contact (tBIC) were measured.

Histological analysis was performed using a light microscope (BX51, OLYMPUS, Tokyo, Japan) connected to a CCD camera (SPOT Insight 2 Mp scientific CCD digital Camera system, DIAGNOSTIC instruments, Inc, USA) and an adaptor (U-CMA3, OLYMPUS, Japan). Histologic observation was performed at a magnification of 10x, and histomorphometric measurements were performed at 30 × magnification. Two implants in 3D-none, two in 3D-SLA, and two in 3D-TIPS were inserted bicortically in the coronal and apical regions. However, most were placed monocortically in the coronal region. Therefore, two regions of interest (ROI) with a width of 3 mm and a length of 2 mm based on the center of the 3D printed implants at right and left were set 2 mm from the superior margin of the lattice area of the implants (Fig. 6A). Histomorphometric measurements were conducted twice by a blinded, experienced examiner (J.-B.L) using image analysis software (ImageJ Version 1.53a, National Institutes of Health, USA). The implants were demarcated with inner and outer areas at the region of interest (ROI) due to the lattice structure of the 3D-printed implants. The following parameters were measured according to a previous study^11^: mineralized bone-to-implant contact (mBIC), osteoid-to-implant contact (OIC), total bone-to-implant contact (tBIC), mineralized bone area fraction occupancy (mBAFO), osteoid area fraction occupancy (OAFO), and total bone area fraction occupancy (tBAFO) in the inner and outer areas of the lattice structure of the 3D-printed implants at the region of interest (Fig. 6B,C).

### Statistical analysis

Sample size calculation was performed based on a previous study^5^ using G*power (version 3.1., Autenzell, Germany). Type I error was set at 0.05, and type II error was set at 0.2. The clinically relevant difference was set at 20% of mean BIC with a standard deviation of 5%. Host responses to the placed 3D-printed implants can be affected by systemic conditions. Therefore, a sample size of nine animals was calculated per experimental group considering a 10% dropout rate, regarding each animal as a statistical unit. Three different surfaces of implants were placed in each animal, and one of them was placed additionally.

Statistical analyses were performed with SPSS version 19 software (IBM Software, Armonk, NY, USA). After rejecting the normality assumption performed by Shapiro–Wilk’s test, a nonparametric statistical method was used. Kruskal–Wallis tests were used to determine the level of significance accompanied by a Bonferroni multiple comparison with the Mann–Whitney test. The difference was considered significant when the P value was < 0.05.

Intra‐examiner reliability for the histomophometric measurements was calculated using the inter‐class correlation coefficient. The inter‐class correlation coefficient was 0.988 with a 95% confidence interval of 0.954–0.997 indicating high intra‐examiner reliability (p < 0.001).

## Supplementary Information

Supplementary Information 1.

Supplementary Information 2.
