# Supplementary material for: The impact of surface treatment in 3-dimensional printed implants for early osseointegration: a comparison study of three different surfaces
Source: Sci Rep. 2021 May 17;11:10453. doi: 10.1038/s41598-021-89961-3 (PMC8129142; doi:10.1038/s41598-021-89961-3)
Supplement: Supplementary file 1 — Supplementary Information 1. [file 41598_2021_89961_MOESM1_ESM.docx]

Supplement 1. The processing parameters for 3D printing implants

TiGr2: Titanium grade 2

| Ti Gr2 | 66.67 | 180 | 1500 | 0.06 | 0.03 | 15 | 220 | 0.07 |
| --- | --- | --- | --- | --- | --- | --- | --- | --- |

| **Powder Type** | **Energy Density**  **E (J/mm^3^)** | **Laser Power**  **P (W)** | **Scanning Speed**  **v (mm/s)** | **Hatching Distance**  **h (mm)** | **Layer Thickness**  **t (mm)** | **Gas Flow (%)** | **Dose Factor (%)** | **Offset to Original Contour (mm)** |
| --- | --- | --- | --- | --- | --- | --- | --- | --- |

**Supplement 2.** Surgical procedure for the placement of 3D printed implants in rabbit tibia model. (a) Full thickness flap elevation. (b) Two sites were prepared for 3D printed implant placement in each tibia. (c) Malleting was performed for placement of two implants. (d) Two 3D printed implants were placed in each tibia. (e) Subcutaneous sutures were performed. (f) Primary closure of skin was performed.
